# The Practical Training of the Royal Army Medical Corps

**Published:** 1913-06-21

**Authors:** 


### THE PRACTICAL TRAINING OF THE ROYAL ARMY MEDICAL CORPS.

One of the outstanding features of the Aldershot Army Manœuvres last autumn was the very interesting demonstration given in the vicinity of Frensham by the R.A.M.C. of the modern system of succouring the wounded on the field of battle. Its purpose was no doubt the double one of testing the efficiency of the medical units concerned and also of furnishing a practical illustration of the great improvements which have been carried out in the Medical Service of the Army since the South African War. To enable the demonstration to take place there were brought together in the Aldershot command three field ambulances and a clearing hospital, all of them on a war establishment. This "mobilisation," or bringing them up to war strength, was accomplished by drawing officers and other ranks from Netley and elsewhere to reinforce the local personnel. When so completed, the field ambulances, which are the mobile portion of the Medical Service of a Division, were posted to General Lawson's Division, and the clearing hospital was established at Aldershot. The demonstration was carried out by General Lawson's Division taking the field and operating in the Woolmer Forest country against other troops stationed in that part of the command, the plan of operations being so devised that the fighting would be accompanied by heavy casualties, corresponding in number to what might be reasonably expected in actual warfare, and placed in selected situations, so that they might test the resources and the practical skill of the Medical Service.

In dealing with these Divisional casualties, the work of the R.A.M.C. was confined to the actual tending of the wounded in the firing line and to their transference to a clearing hospital in the rear, so that the assistance given included (1) the rendering of first aid by the regimental stretcher-bearers; (2) removal by the stretcher squads of the Bearer Division of the Field Ambulance; (3) attention at the dressing station on the field; (4) removal by horsed ambulance to Tent Division of the Field Ambulance for surgical treatment; and, lastly (5), conveyance to the clearing hospital. This accomplished, nothing further was done, no steps being taken to move the cases from the clearing hospital by ambulance train to a stationary or a general hospital.

As regards the medical personnel provided for the care of the wounded of a Division, it is made up of the regimental stretcher-bearers, who have a total strength of about 260 or more, and of the three Field Ambulances, each of which numbers ten officers, 240 other ranks, twenty-two vehicles, and 100 horses, and is composed of a Bearer Division for collecting the wounded, and of a Tent Division for furnishing the necessary immediate surgical treatment required. In each Bearer Division are eighteen stretcher squads of six men each, and attached to it are ten ambulance waggons, with four horses each, every waggon being able to carry four cases lying down or twelve sitting up, or two lying down and four sitting up. There are, in short, available for the care of and the removal from the fighting line of the casualties of a Division some 260 or more regimental stretcher-bearers, the fifty-four stretcher squads, numbering 324 men of the Bearer Division, and thirty horsed ambulance waggons carrying, on the average, 200 casualties. Close behind are the three Tent Divisions of the Field Ambulances ready to give any immediate surgical treatment needed, and able to provide tent accommodation for 450 serious cases.

In the Aldershot exercises the mode of procedure followed was that during the fighting certain "casualties" were provided, being picked out from the different units by the company officers or by officers of the umpire staff. The officers and men selected as "wounded" had affixed to them "tabs" on which were stated the nature of the supposed "wound" that had been received, and the instructions were that each casualty was to be dealt with as if the supposed injury had really been received. Accordingly, the regimental stretcher-bearers gave the required "first aid" in the shape of binding up the wound, arresting bleeding, and providing temporary shelter, and then the stretcher squads carried out the necessary removal to the "dressing stations." These latter were got ready in all details, being prepared for the performance of absolutely urgent operations just as they would be in the case of a genuine battle. The clearing hospital being located in Aldershot some miles in rear of the fighting area, the problem of removing the wounded from the Tent Divisions into it was solved by utilising the motor lorries belonging to the Transport of the

Division, as would probably be done in time of war. All who witnessed the work done during the two days over which the demonstration extended were impressed by its excellence and by its thoroughness, and were unanimous in the opinion that there has been most creditable progress in the training and efficiency of the R.A.M.C. Much has been done in the past through the efforts of able administrators, such as the late Director-General, Sir Alfred Keogh, to make the Army medical officer that necessary combination of scientific acquirements and practical knowledge of his work that his duties imperatively demand; but a medical officer, no matter how high his attainments, is placed under great disadvantages if the R.A.M.C. to which he belongs is hampered, or it may be paralysed, in its sphere of work by want of independence. That this may happen under existing arrangements is quite a possible thing, and as an illustration of it may be given the conditions of its transport supply. These are not as they should be. At present the R.A.M.C. is dependent on the Army Service Corps for horses to give its ambulances mobility; but if these horses are not forthcoming, as has happened in the past, the system of succouring the wounded at once breaks down, and the battle area will not be cleared with the celerity that marked the recent demonstration. This state of matters should be altered at once, and the R.A.M.C. should have its own transport drivers and horses just as the Territorial field units have. In this way only can it become the self-contained and independent corps that it should be; and strong pressure should be brought to bear on the authorities to concede this most essential reform.

As carried out, these recent exercises could not be but of great value to all ranks of the R.A.M.C., and we should like to see similar ones for practising the conveyance of the wounded from the clearing hospital by ambulance train to the base, too little attention being given in our opinion to this train work. We also feel that the demonstration has its lessons for those in authority, for it points clearly to the need of an improved ambulance waggon, if possible, in the shape of a motor vehicle; and we hope the matter will have the attention it deserves. Further, a display such as this brings home to everyone very vividly the heavy demands that will be made upon the R.A.M.C. were the Expeditionary Force to be suddenly mobilised. In view of this, one naturally asks if there is ready the trained personnel and the medical and surgical equipment to meet the needs of all the Divisions of that Force. We hope that both can be at once provided; but if it is the case that it cannot be, no time should be lost in providing it, for no matter how perfect a system is it will fail absolutely if there are not competent men to work it; and we do not want repeated the unfortunate episodes of the South African War.

